# Isolation, Characterization and Immunomodulatory Activity Evaluation of Chrysolaminarin from the Filamentous Microalga *Tribonema aequale*

**DOI:** 10.3390/md21010013

**Published:** 2022-12-24

**Authors:** Feifei Wang, Rundong Yang, Yuhao Guo, Chengwu Zhang

**Affiliations:** 1 School of Modern Industry for Selenium Science and Engineering, Wuhan Polytechnic University, Wuhan 430023, China; 2 School of Food Science and Pharmaceutical Engineering, Nanjing Normal University, Nanjing 210023, China; 3 Department of Ecology and Research Center for Hydrobiology, Jinan University, Guangzhou 510632, China

**Keywords:** *Tribonema*, chrysolaminarin, isolation, immunomodulatory activity

## Abstract

The aim of this study is to investigate the differences in the accumulation capacity of chrysolaminarin among six *Tribonema* species and to isolate this polysaccharide for immunomodulatory activity evaluation. The results showed that *T. aequale* was the most productive strain with the highest content and productivity of chrysolaminarin, which were 17.20% (% of dry weight) and 50.91 mg/L/d, respectively. Chrysolaminarin was then extracted and isolated from this alga, and its monosaccharide composition was mainly composed of a glucose (61.39%), linked by β-D-(1→3) (main chain) and β-D-(1→6) (branch chain) glycosidic bonds, with a molecular weight of less than 6 kDa. In vitro immunomodulatory assays showed that it could activate RAW264.7 cells at a certain concentration (1000 μg/mL), as evidenced by the increased phagocytic activity and upregulated mRNA expression levels of *IL-1β*, *IL6*, *TNF-α* and *Nos2*. Moreover, Western blot revealed that this polysaccharide stimulated the phosphorylation of p-65, p-38 and JNK in NF-κB and MAPK signaling pathways. Overall, these findings provide a reference for the further development and utilization of algae-based chrysolaminarin, while also offering an in-depth understanding of the immunoregulatory mechanism.

## 1. Introduction

Microalgae-based biodiesel has attracted much attention because it is renewable, sustainable and environmentally friendly, but its production cost is too high to compete with fossil fuels [1,2]. To improve its economic feasibility, substantial efforts are underway to establish an integrated biorefinery process aimed at maximizing the optimization of algal biomass to coproduce biodiesel and high-value bioproducts such as carotenoids, polyunsaturated fatty acids, active polysaccharides, etc. [3,4]. These could be employed for various practical uses due to their distinctive properties.

Microalgae contain abundant quantities of natural polysaccharides owing to their enormous biodiversity [5], and many studies have reported that microalgal polysaccharides have a wide range of biological activities, including antioxidant, antitumor, anti-inflammatory and immunostimulatory properties [6,7,8]. In particular, some algal polysaccharides containing sulfate esters exhibit unique pharmacological activities due to their novel and complex structure, including sulfated polysaccharides p-KG03 extracted from *Gyrodinium impudium* [9], which show specific antiviral activity by inhibiting the processes of virus–cell interaction and virus–cell fusion at the same time. Therefore, microalgal polysaccharides are regarded as valuable new bioactive compounds with many downstream applications in the food, cosmetics, neutraceutical and pharmaceutical industries [10].

Polysaccharides have various important biological functions in algal cells, including storage, protection and structural roles [11]. Among them, chrysolaminarin is a class of principal energy-storage polysaccharides that is widely distributed in diatoms and chrysophites [12]. In many diatoms, chrysolaminarin is a soluble and low-molecular-weight β-glucan (1–40 kDa) consisting of glucose monomers linked by β-1,3 bonds with limited β-1,6 branches [13], but its molecular weight, monosaccharide composition and number of branches are species-specific. Chrysolaminarin isolated from microalgae showed some interesting bioactivities, such as the scavenging of hydroxyl radicals and antitumor activity [14,15]. Thus, interest in the selection and cultivation of chrysolaminarin-rich microalgae strains has increased, especially with regard to those that can accumulate lipids and chrysolaminarin simultaneously.

*Tribonema* spp. are filamentous oleaginous microalgae belonging to the class Xanthophyceae [16]. In the past, most studies have focused on culturing *Tribonema* microalgae for biodiesel and palmitoleic acid production, owing to their high content of lipid and palmitoleic acid, high resistance to grazer predation and ease of harvest, so they have been considered an emerging potential biorefinery biomass feedstock for the co-production of bioenergy and valuable bioproducts to improve economic efficiency [17,18]. However, little attention has been paid to the fact that *Tribonema* spp. are also rich in active polysaccharides [6,19], such as a sulfated polysaccharide isolated from *Tribonema* spp. that shows anticancer and immunomodulatory activities. In addition, chrysolaminarin is an important intracellular polysaccharide in *Tribonema* species that is responsible for energy storage [20]. However, little is known about the structure of chrysolaminarin isolated from *Tribonama* microalgae and its immunoregulatory activity. In addition, the abilities of these species to accumulate chrysolaminarin are different and species-specific, but have been poorly researched. In this context, we first compared the differences in chrysolaminarin production ability of six *Tribonema* species under photoautotrophic conditions and then extracted, isolated and characterized this polysaccharide from the most productive microalga. The immunoregulatory activity of isolated chrysolaminarin was also evaluated in vitro. The key aim of the present study is to provide new insights into the accumulation, isolation and immunoregulatory activity of chrysolaminarin in *Tribonema* and to broaden the potential applications of microalgae-based products.

## 2. Results and Discussion

### 2.1. Comparison of Chrysolaminarin Production in Six Tribonema Species

Chrysolaminarin is known as the primary assimilative product of the genus *Tribonema* [21], but few studies have investigated differences in the ability to accumulate this compound. Therefore, six *Tribonema* species were cultured in mBG-11 medium to screen for a chrysolaminarin-producing algal strain.

**Figure 1:**
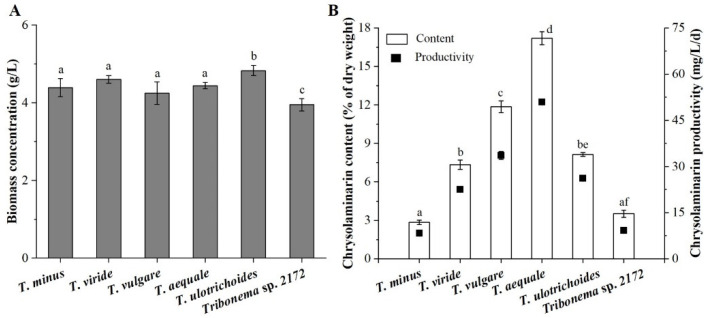
Biomass (**A**) and chrysolaminarin production (**B**) of six *Tribonema* species in normal mBG-11 medium. Different lowercase letters above the bars indicate significant differences at *p* < 0.05. Values are expressed as the means ± SD of three replicates.

As shown in Figure 1A, the algal strains grew well in mBG-11 medium with little difference in biomass, with the exception of *T. ulotrichoides*, which had the highest biomass at 4.83 ± 0.13 g/L. Nevertheless, *T. aequale* showed the highest content of chrysolaminarin (17.20% ± 0. 51% of dry weight), followed by *T. vulgare* (11.86% ± 0.45% of dry weight), *T. ulotrichoides* (8.13% ± 0.14% of dry weight) and *T. viride* (7.34% ± 0.37% of dry weight) (Figure 1B). The amount of chrysolaminarin in *T. aequale* was approximately six times higher than that in *Tribonema* sp.2172 and *T. minus*, which revealed that the ability of the tested algal strains to accumulate chrysolaminarin was species-specific. Based on observation of the accumulation biomass and chrysolaminarin in six *Tribonema* species, the maximum chrysolaminarin productivity was found in *T. aequale*, which reached 50.91 mg/L/d. Thus, this strain was selected and used as biomass feedstock for further extraction and isolation of chrysolaminarin in the subsequent studies.

### 2.2. Preparation and Characterization of Chrysolaminarin from T. aequale

**Figure 2:**
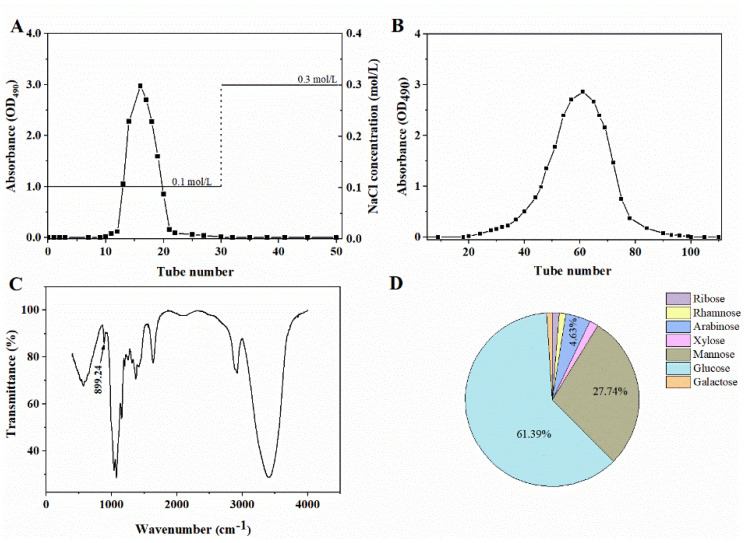
Isolation and structural characterization of chrysolaminarin from *Tribonema aequale*. (**A**) DEAE-52 anion-exchange chromatography; (**B**) Sephadex G-200 gel-filtration chromatography; (**C**) FT-IR spectra; (**D**) monosaccharide composition.

Crude polysaccharide was extracted from *T. aequale* biomass with a yield of 0.17 g/g algal powder, which was separated by DEAE-52 column and eluted with 0.1 mol/L NaCl, and the target elution peak was obtained as shown in Figure 2A. After further isolation by a Sephadex G-200 column with 0.1 mol/L NaCl eluting, a single main fraction (the isolated chrysolaminarin, Figure 2B) was then obtained at a yield of 0.11 g/g algal powder and used for further analysis.

As shown in Figure 2C, the FT-IR spectra of isolated chrysolaminarin displayed some absorption peaks at 3406, 2921 and 1400–1200 cm^−1^, which are the typical absorption peaks of polysaccharides [22,23]. The stretching peaks at 1637.7 cm^−1^ and 1374.7 cm^−1^ represent the carboxyl groups [15]. Most importantly, a characteristic absorption peak at 889.24 cm^−1^ was attributed to the presence of β-type glycosidic linkages [15,22,24].

To understand the monosaccharide composition and molecular weight of chrysolaminarin isolated from *T. aequale*, GC−MS and HPGPC were used in this study. As shown in Figure 2D, its monosaccharide composition was mainly composed of ribose (1.32%), rhamnose (1.24%), arabinose (4.63%), xylose (1.62%), mannose (28.74%), glucose (61.39%) and galactose (1.06%) according to the GC-MS analysis. Glucose accounted for the largest proportion in total sugars, followed by mannose, which was similar to the monosaccharide composition of chrysolaminarin isolated from *T. utriculosum* [20]. However, Xia et al. [15] reported that the content of glucose in chrysolaminarin isolated from *Odontella aurita* reached 82.23%. This difference might be due to species specificity. Meanwhile, the isolated chrysolaminarin had a number-average molecular weight (Mn) of 5.24 kDa and a weight-average molecular weight of 5.99 kDa based on calibration with standard dextrans, and the degree of dispersion (Mw/Mn) was 1.14. The results showed that chrysolaminarin isolated from *T. aequale* was a heteropolysaccharide with low molecular weight, similar to that isolated from *Phaeodactylus tricornutum* [22].

**Figure 3:**
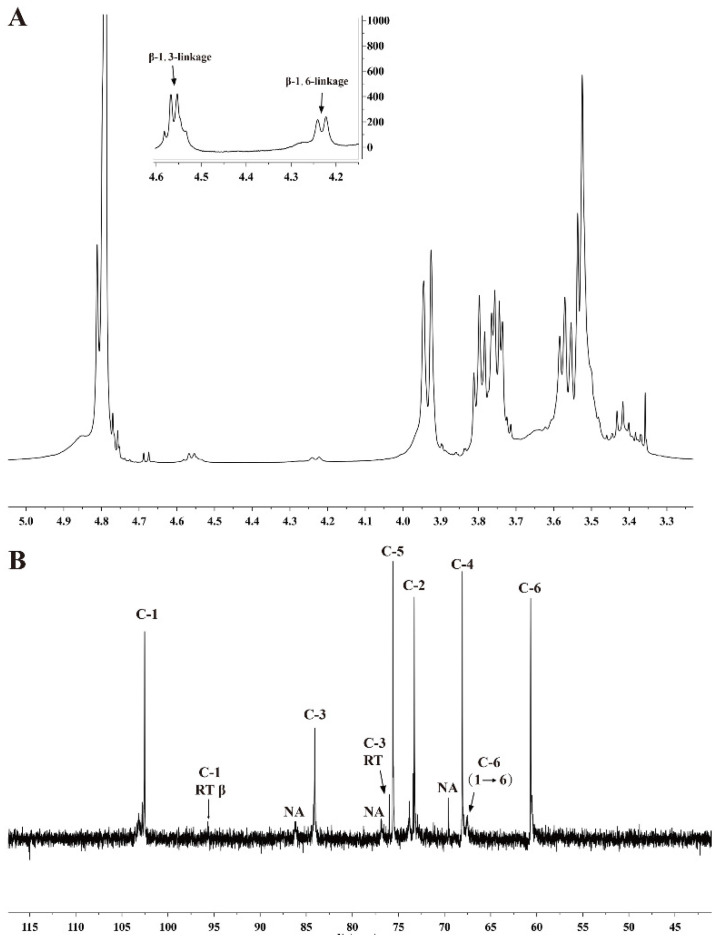
NMR spectra of chrysolaminarin isolated from *T. aequale*. (**A**) ^1^H NMR spectra; (**B**) ^13^C NMR spectra; NA, not assigned.

In addition, in the ^1^H NMR spectra of the isolated chrysolaminarin (Figure 3A), two anomeric proton singles at δ 4.58 and 4.24 ppm confirmed the presence of β-type glycosidic linkages, which were assigned to H-1 of the β-1,3-linkage and β-1,6-linkage, respectively [22]. These were in accordance with the aforementioned FT-IR results. The ^13^C NMR spectra showed major signals at δ 102.4–102.6, 84.0–84.2, 76.0, 75.4–75.7, 73.2–73.3, 67.9–68.1, 67.4–67.7 and 60.3–60.7 (Figure 3B), especially the anomeric carbon with a signal at δ102.5 ppm, which indicated that the glucosyl-linkage was β-type. These results were consistent with that chrysolaminarin isolated from *Odontella aurita* [15] and *P. tricornutum* [22]. Thus, the isolated polysaccharide was unambiguously identified as a chrysolaminarin with a β-D-(1→3)-(main chain) and a β-D-(1→6) (branch chain)-linked glucopyranan structure.

### 2.3. Effects of Isolated Chrysolaminarin on Macrophages Viability and Phagocytic Activity

**Figure 4:**
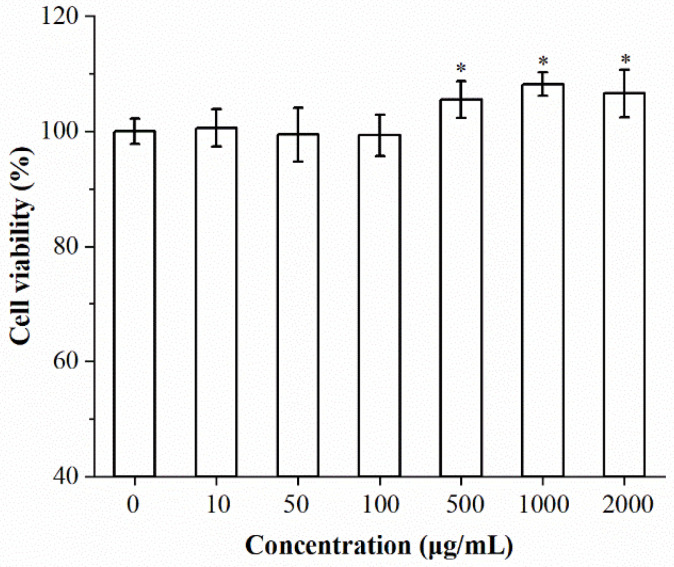
Effect of isolated chrysolaminarin on the viability of RAW264.7 cells. Values are expressed as the means ± SD of three replicates, and significant differences from control group are indicated by * *p* < 0.05.

The cytotoxic effect of isolated chrysolaminarin on RAW264.7 cells was investigated by using the MTT assay. As shown in Figure 4, the isolated chrysolaminarin was nontoxic to macrophages within the tested concentration (10–2000 μg/mL), and it also exerted growth-promoting effects in the concentration ranging from 500 to 2000 μg/mL.

**Figure 5:**
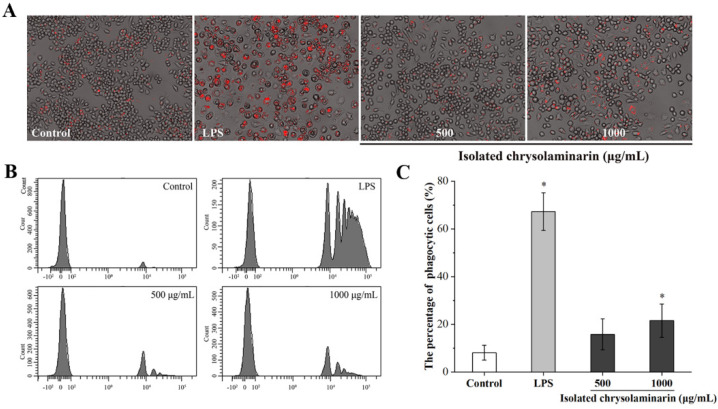
Effect of isolated chrysolaminarin on phagocytic activity of RAW264.7 cells. (**A**) The pictures of macrophages engulfing fluorescent latex beads under a fluorescence microscope; (**B**) phagocytosis assay was examined by flow cytometry; (**C**) the percentage of phagocytic cells is determined by the number of macrophages that ingest at least one fluorescent latex bead divided by 1000 macrophages. Significant differences from control group are indicated by * *p* < 0.05. The data are presented as mean ± SD (*n* = 3).

Phagocytosis is a basic cellular process that plays a crucial role in immunity, and the phagocytosis ability of macrophages can indirectly reflect the level of their immune activity [25,26]. Therefore, the effect of isolated chrysolaminarin on the phagocytic activity of RAW 264.7 cells was determined based on the uptake of fluorescent latex beads and analyzed using immunofluorescence microscopy and flow cytometry. As shown in Figure 5A, it was obvious that the isolated chrysolaminarin treatment leads to an increase in phagocytic activity in RAW 264.7 cells, as evidenced by the red fluorescent dots observed inside the chrysolaminarin-treated cells by microscopic analysis. This effect is further demonstrated by flow cytometry analysis, in which more phagocytic fluorescent latex beads were detected on the histogram after treatment with isolated chrysolaminarin compared to the control (Figure 5B). In addition, with the increase in concentration from 500 to 1000 μg/mL, the percentage of phagocytic cells increased from 15.8 to 21.5% (Figure 5C), while the latter was significantly different from the control group (*p* < 0.05). Although the phagocytic activity of isolated chrysolaminarin was obviously lower than that of the positive control (LPS), these results indicated that the appropriate concentration of isolated chrysolaminarin could activate macrophages to enhance its phagocytic activity.

### 2.4. Effects of Isolated Chrysolaminarin on mRNA Expression of Selected Cytokines

**Figure 6:**
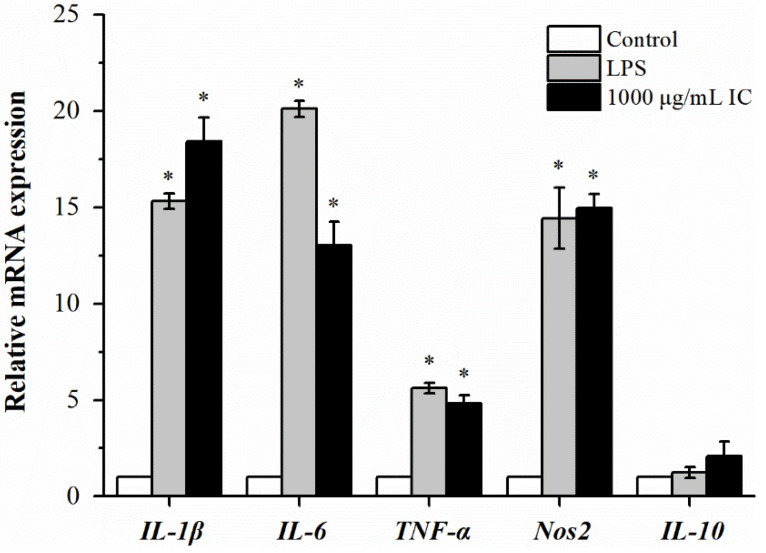
Effects of isolated chrysolaminarin on mRNA expression of selected cytokines (IL-1β, IL-6, TNF-α, Nos2 and IL-10) in RAW264.7 cells. Data are expressed as the mean fold change (mean ± SE, *n* = 3) from the calibrator group (Control). The concentration of chrysolaminarin to stimulate RAW264.7 cells is 1000 μg/mL. IC, isolated chrysolaminarin. Significant differences from control group are indicated by * *p* < 0.05.

As a typical β-glucan, chrysolaminarin and its extracts are considered to be an immune booster through the activation of macrophages and the production of proinflammatory cytokines [12]. Among them, TNF-α can kill tumor cells or suppress their growth and enhance the phagocytosis, proliferation and differentiation of neutrophils; IL-1β can activate immune cells, assist in T-cell proliferation, participate in the production of antibodies and promote inflammation; IL-6 is the major factor that mediates inflammation, as it can activate immune cells to exert an immunoregulatory effect [27]. Therefore, the effects of isolated chrysolaminarin (1000 μg/mL) on stimulating RAW264.7 cells to secrete cytokines was determined at the mRNA expression level through RT-PCR and is shown in Figure 6. As expected, compared to the control group, the isolated chrysolaminarin had the same effect as LPS (positive control), which could significantly upregulate mRNA expression levels of *IL-1β*, *IL6*, *TNF-α* and *Nos2*, with the exception of *IL10*. Moreover, it even obtained a higher mRNA expression of *IL-1β* than LPS. Zou et al. [28] reported that cytokine IL-1β acts as a mediator of β-glucan actions and triggers a generalized downstream response through the NF-κB and MAPK signaling pathways to produce cytokines and activate the migration and phagocytic activities of macrophages. In addition, *Nos2* is responsible for controlling NO synthesis, which is an indispensable immunoregulator involved in multiple physiological and pathological processes related to immune response [29]. Figure 6 showed that mRNA expression level of *Nos 2* in RAW 264.7 cells treated with 1000 μg/mL of isolated chrysolaminarin was comparable to that of the positive group (LPS, 500 ng/mL), indicating that more NO may be produced to improve the activation state of macrophages. In summary, the isolated chrysolaminarin at a certain concentration effectively upregulated the mRNA expression of *TNF-α*, *IL-6*, *Nos2* and *IL-1β* in RAW 264.7 cells, promoted the release of cytokines and NO, and thus exerted an immunoregulatory effect.

### 2.5. Effects of Isolated Chrysolaminarin on the MAPK and NF-κB Signaling Pathways

**Figure 7:**
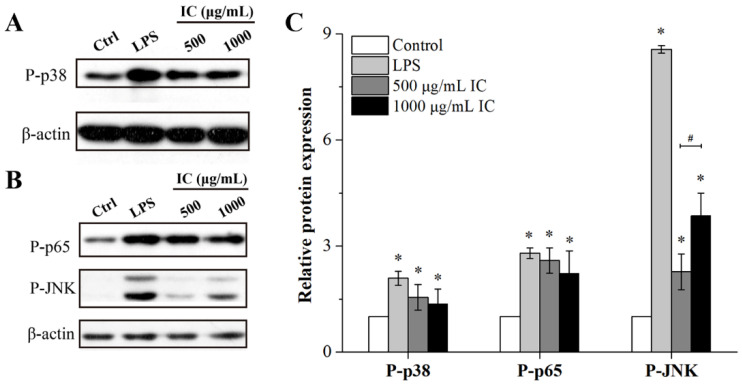
Effects of isolated chrysolaminarin on phosphorylation of key proteins in MPAK and NF-κB signaling pathways in RAW 264.7 cells. (**A**,**B**) Representative blot images of phosphorylation levels of p38, p65 and JNK; (**C**) the quantified expression of P-p38, P-p65 and P-JNK compared with controls, which were corrected to protein β-actin (internal control). IC, isolated chrysolaminarin. * *p* < 0.05 versus the control group; ^#^ *p* < 0.05, the groups treated with different concentrations of isolated chrysolaminarin only. The data are presented as mean ± SD (*n* = 3).

MAPK and NF-κB signaling pathways are known to be involved in the production of cytokines in response to various stimuli [25]. In this study, high mRNA expression levels of *IL-1β*, *IL6*, *TNF-α* and *Nos2* were observed in RAW 264.7 cells treated with isolated chrysolaminarin, but the mechanism of action remains unclear. Many studies have reported that plant polysaccharides could activate macrophages through triggering phosphorylation within MAPK (including p38, ERK and JNK) and NF-ĸB (p65) signaling pathways, thereby promoting the secretion of TNF-a, IL-6 and NO in RAW264.7 macrophages [25,30]. Therefore, the effects of isolated chrysolaminarin on the phosphorylation of key proteins in signaling pathway such as p38, JNK and p65 were assessed through Western blot in order to investigate the mechanism underlying the activation of macrophages. As shown in Figure 7A,B, the isolated chrysolaminarin stimulated RAW 264.7 cells with the similar effect to LPS (positive control), both of which increased the protein levels of P-p38 and P-JNK proteins in the MAPK signaling pathway. In particular, when the concentration increased from 500 μg/mL to 1000 μg/mL, the induced phosphorylation of JNK was concentration-dependent, which was 2.27 and 3.85 times higher than that of the control, respectively (Figure 7C). In addition, the phosphorylation of p65 is a vital step for activating the NF-ĸB signaling cascade [31], and the isolated chrysolaminarin was found to induce the phosphorylation of p65 (Figure 7B,C), suggesting that the NF-ĸB signaling pathway was also involved in the immune enhancement effect. Above all, the results showed that the phosphorylation levels of p38, JNK and p65 could be increased by a certain concentration of isolated chrysolaminarin, indicating that the MAPK and NF-ĸB signaling pathways were closely associated with macrophage activation.

## 3. Materials and Methods

### 3.1. Materials

The standard monosaccharides (glucose, xylose, galactose, rhamnose, mannose and arabinose), lipopolysaccharide (LPS, *Escherichia coli* 055:B5) and T-series Dextran standards (1–670 kDa) were purchased from Sigma-Aldrich Co. (Shanghai, China). Penicillin–streptomycin solution, dimethyl sulfoxide (DMSO) and 3-(4,5-dimethylthiazol-2-yl)-2,5-diphenyltertrazolium bromide (MTT) were purchased from Solarbio Co. (Beijing, China). DMEM was purchased from Sangon Biotech Co., Ltd. (Shanghai, China). DEAE-52 cellulose and Sephadex G-200 were purchased from Guangzhou Pengcheng Biotechnology Co., Ltd. (Guangzhou, China). The solvents and other chemicals used were analytical-grade.

### 3.2. Microalgae Strains and Culture Conditions

The six *Tribonema* species (*Tribonema* sp.2172, *T*. *ulotrichoides*, *T*. *viride*, *T. minus*, *T. aequale* and *T*. *vulgare*) used in this study were purchased from the Culture Collection of Algae at the University of Göttingen (SAG). All stock cultures were maintained in modified BG-11 (mBG-11) medium [32] and deposited in our laboratory.

The starter cultures of six *Tribonema* species were separately prepared in a Ø6 × 60 cm (inner diameter × length) glass column photobioreactor containing 1.2 L of mBG-11 medium and grown under low-light conditions at 70–80 μmol/m^2^/s and 1% CO_2_ (*v*/*v*) agitation. After 7 days of cultivation, the algal cells were collected by filtration, washed twice with sterile water and then used as seed cultures to inoculate into Ø6 × 60 cm glass column photobioreactors for cultivation at the same initial cell density of 0.35–0.40 g/L. These microalgae were cultured in mBG-11 medium with continuous unilateral light illumination of 300 ± 15 μmol/m^2^/s and bubbled with 1% CO_2_ (*v*/*v*) from the bottom of the column for 15 days. At the end of cultivation, the culture samples were harvested by filtration using bolting silk (300 mesh) and then lyophilized in a vacuum freeze drier (Christ, Germany). The algal powder was stored at 4 °C prior to analysis.

### 3.3. Determination of Biomass Dry Weight and Chrysolaminarin Content

Biomass dry weight was measured on day 15 according to Wang et al. [33]. Briefly, 10 mL of cultures was filtered through 0.45 μm preweighted GF/B filter paper (DW_0_), and dried at 105 °C overnight to a constant weight (DW_1_). The biomass dry weight (g/L) was calculated as (DW_1_ − DW_0_) × 100. For the determination of chrysolaminarin content, it was extracted from the algal powder (50 mg) with diluted sulfuric acid (50 mmol/L) and then quantitatively assayed using the phenol–sulfuric acid method, as detailed by Xia et al. [15].

### 3.4. Extraction and Isolation of Chrysolaminarin

The lyophilized algal sample (40 g) was first mixed with 1 L of diluted sulfuric acid (50 mmol/L), extracted by acidolysis in a water bath at 80 °C and pretreated with ultrasound. The supernatant was collected after centrifugation, and the crude polysaccharide was then obtained by the processes of alcohol precipitation, deproteinization and dialysis, which have been described in detail by Xia et al. [15] and Zhang et al. [22]. Subsequently, the treated crude polysaccharide was redissolved in deionized water and loaded on a pre-equilibrated DEAE-cellulose-52 (3 × 30 cm) column. The column was gradient-eluted with 0.1 and 0.3 mol/L NaCl solution at a flow rate of 2 mL/min. Each 5 mL of eluate was collected as a tube to detect the variation in polysaccharide concentration using the phenol–sulfuric acid method [22]. Afterwards, chrysolaminarin was further purified by 0.1 mol/L NaCl solution on a Sephadex G-200 column (2 × 50 cm) with a flow rate of 2 mL/min. The corresponding polysaccharide-rich fraction (2 mL of each tube) was pooled, dialyzed and lyophilized for further analysis.

### 3.5. Characterization of Chrysolaminarin

For Fourier transform infrared spectroscopy (FT-IR) (Thermo Fisher Nicolet iS50, Waltham, MA, USA) analysis, the sample (2 mg) was first mixed with the dried KBr (*w*/*w* = 1:100), and then pressed into a flake for measurement. The spectrum was recorded in the range of 500–4000 cm^−1^ at a resolution of 2 cm^−1^. The molecular weight of the isolated chrysolaminarin was measured by high-performance gel permeation chromatography (HPGPC) (Agilent 1260, Santa Clara, CA, USA) equipped with a TSK-G3000 PW_XL_ column (7.5 mm × 300 mm) and a refractive index detector (Agilent RID-10A Series) [20]. The molecular weight was estimated by reference to a calibration curve made with T-series Dextran standards (1–670 kDa). The monosaccharide composition was determined using gas chromatography–mass spectrometry (GC–MS) as described by Xia et al. [15]. Identification of the derivatized monosaccharide was carried out according to the retention time and mass fragmentation patterns of the standards. For nuclear magnetic resonance (NMR) analysis (Bruker Advance III 500, Karlsruhe, Germany), the sample (10 mg) was dissolved in D_2_O (deuterium oxide) in an NMR tube, and the ^1^H (500 MHz) and ^13^C (125 MHz) spectra were recorded at 30 °C.

### 3.6. Immunomodulatory Activity In Vitro

#### 3.6.1. Cell Culture

Murine RAW 264.7 (ID: TIB-71) macrophage cells were purchased from the Cell Bank of the Chinese Academy of Science (Shanghai, China) and cultured in DMEM supplemented with 10% FBS and 1% penicillin-streptomycin in an anaerobic incubator with 5% CO_2_. Treatments included normal group (control), LPS group (positive control, LPS) and different concentrations of isolated chrysolaminarin. In addition, the endotoxin content was estimated to be less than 0.015 EU/mg using endotoxin-specific kit (Chinese Horseshoe CrabReagent Manufactory, Co., Xiamen, China). For most experiments, cells were allowed to adhere for 24 h before treatment.

#### 3.6.2. RAW 264.7 Cell Viability Assay

Cell viability was evaluated using the MTT method according to Palanisamy et al. [34]. In brief, cells were seeded in a 96-well plate at a density of 5 × 10^4^ cells/mL and exposed to different concentrations of isolated chrysolaminarin (10, 50, 100, 500, 1000 and 2000 μg/mL) for 24 h, then MTT solution was added to each well and incubated for another 4 h. After removal of the supernatant, 100 μL of DMSO was added to promote crystal dissolution. The absorbance at 490 nm was measured using a microplate reader (ELX800, BioTek, Winooski, VT, USA), and the percentage of cell viability was calculated as follows:

Cell viability (%) = (OD_490_ value of treated cells/OD_490_ value of control cells) × 100.

#### 3.6.3. Phagocytosis Assay

Phagocytic activity was determined by the amount of fluorescent-labeled latex beads engulfed by RAW 264.7 cells as described previously [35]. In brief, cells were seeded on 6-well plates at a density of 5 × 10^5^ cells/mL, stimulated with isolated chrysolaminarin at the concentrations of 500 and 1000 μg/mL for 24 h and then incubated with fluorescent latex beads (Sigma, L-3030, carboxylate-modified, average size 2 µm, 10 µL beads in 2 mL DMEM medium) for another 4 h. Complete DMEM medium was treated as control group and lipopolysaccharides (LPS, 500 ng/mL) solution was used as the positive control. For flow cytometry analysis, cells were detached by pipetting after washing in PBS, and the fluorescence of 1000 cells per sample was measured by BD FACS Canto flow cytometer (Becton-Dickenson, San Jose, CA, USA). The phagocytic activity was determined by the percentage of phagocytic cells, which meant the number of macrophages that ingest at least one fluorescent latex bead divided by the total number of macrophages. To acquire the pictures of cell-phagocytized fluorescent latex beads, immunofluorescence microscopy (Eclipse Ti-E, Nikon, Tokyo, Japan) was used with TEXAS RED Filter cube.

#### 3.6.4. Quantitative Real-Time PCR for Cytokine Gene Expression in RAW264.7 Cells

RAW264.7 cells were seeded at a density of 5 × 10^5^ cells/mL onto 12-well plates and preincubated in DMEM medium at 37 °C under 5% CO_2_ for 24 h, followed by stimulation treatment with isolated chrysolaminarin (1000 μg/mL) for 12 h. Cells incubated with LPS (500 ng/mL) or DMEM medium were used as the positive control and the blank control, respectively. Next, total RNA was extracted from the treated cells using TRIzol reagent according to the manufacturer’s recommendations, and its quality was evaluated using a spectrophotometer (P330, Implen, Munich, Germany). The cDNAs were synthesized with a PCR instrument (T100 thermal cycler, Bio-Rad, Hercules, CA, USA) using a PrimeScript™ RT Master Mix kit (RR036A, Takara, China) according to the manufacturer’s protocol.

The expression of five selected immune-relevant genes (IL-1β, IL-6, IL-10, Nos 2 and TNF-α) was quantified by real-time PCR (RT-PCR). RT-PCR was performed in a CFX96 Real-time System (Bio-Rad, Hercules, CA, USA) using a SYBR^®^ Premix Ex Taq™ kit (RR420A, Takara, China) in a 25 μL reaction volume, and the specific primers used were as follows: IL-1β, 5′-GAGCCTGTGTTTCCTCCTTG-3′ (forward) and 5′-GTGTTCCTACCCCCAATGTG-3′ (reverse); IL-6, 5′-AGTTGCCTTCTTGGGACTGATG-3′ (forward) and 5′-CAGGTCTGTTGGGAGTGGTATC-3′ (reverse); IL-10, 5′-GTTGCCAAGCCTTATCGGAA-3′ (forward) and 5′-CCGCATCCTGAGGGTCTTC-3′ (reverse); Nos 2, 5′-AAGCAGCTGGCCAATGAG-3′ (forward) and 5′-CCCCATAGGAAAAGACTGCA-3′ (reverse); TNF-α, 5′-GGGAGCAAAGGTTCAGTGAT-3′ (forward), 5′-CCTGGCCTCTCTACCTTGTT-3′ (reverse) and glyceraldehyde-3-phosphate dehydrogenase (GAPDH), 5′-GTCATTGAGAGCAATGCCAG-3′ (forward) and 5′-GTGTTCCTACCCCCAATGTG-3′ (reverse). The reaction mixture and amplification conditions were maintained according to the manufacturer’s instructions. Each sample was tested in triplicate. The melting curve was plotted with GAPDH as the endogenous reference gene, and relative mRNA expression was determined using the 2^−ΔΔCt^ method [27].

#### 3.6.5. Western Blot Analysis

RAW 264.7 cells incubated in the presence of isolated chrysolaminarin or LPS (positive control, 500 ng/mL) for treatment, as described in Section 3.6.3. To investigate the effect of isolated chrysolaminarin on the phosphorylation of p38, JNK and p65 protein related to MAPK and NF-κB signaling pathways, whole cell protein of RAW264.7 cells was extracted with the lysis reagent (P0013G, Beyotime) containing phosphatase inhibitors (P1081, Beyotime), separated on SDS-PAGE (P0012A, Beyotime) and transferred to PVDF membrane (ISEQ00010, Merck Millipore, Darmstadt, Germany). Membranes were treated with 5% skim milk (232100, GBCBIO) for shielding the nonspecific binding site, followed by an incubation with each antibody at 4 °C for overnight. After washing three times with TBST buffer, the strips were incubated with horseradish peroxidase (HRP)-conjoined goat anti-rabbit antibody (1:10,000 dilution, #7074s, Cell Signaling Technology) for 2 h at room temperature. The protein blot was visualized using ECL regents (4AW011-1000, 4A Biotech, Beijing, China). The band intensities were quantified using Image J software (version 1.48, National Institutes of Health, Bethesda, MD, USA). All immunoblot bands were normalized to the intensities of corresponding bands of the internal control (β-actin).

### 3.7. Statistical Analysis

All experiments were performed in triplicate. Numerical data are expressed as the mean ± standard deviation (SD), while the data generated by RT-PCR are presented as the means of three biological replicates ± standard error (SE). One-way analysis of variance (ANOVA) was followed by Student–Newman–Keus tests using a statistical analysis software package (GraphPad Prism ver. 8, La Jolla, CA, USA) and statistically significant differences were defined as *p* < 0.05.

## 4. Conclusions

This study showed that chrysolaminarin accumulation ability of different *Tribonema* strains was species-specific, and *T. aequale* had the highest chrysolaminarin production. Chrysolaminarin was then isolated from this alga, and structural analysis indicated that it was a low-molecular-weight heteropolysaccharide with a high proportion of glucose, mainly linked by β-D-(1→3) (main chain) and β-D-(1→6) (branch chain) glycosidic bonds. In vitro immunoregulatory assays showed that it could activate RAW 264.7 cells, increase their phagocytic activity, upregulate mRNA expression levels of *IL-1β*, *IL-6*, *IL-10* and *Nos 2* and induce the phosphorylation of target proteins in MAPK and NF-κB signaling pathways. This study represents the first report on the isolation, characterization and immunomodulatory activity of chrysolaminarin from *T. aequale*, which provides a reference for further development and in-depth understanding of its immunoregulatory mechanism.

## Data Availability

The data presented in this study are available on request from the corresponding author. The data are not publicly available due to these data also form part of an ongoing study.
